# New cytogenetically visible copy number variant in region 8q21.2

**DOI:** 10.1186/1755-8166-4-1

**Published:** 2011-01-05

**Authors:** Marina Manvelyan, Friedrich W Cremer, Jeannette Lancé, Rüdiger Kläs, Christina Kelbova, Christian Ramel, Herbert Reichenbach, Catharina Schmidt, Elisabeth Ewers, Katharina Kreskowski, Monika Ziegler, Nadezda Kosyakova, Thomas Liehr

**Affiliations:** 1 Jena University Hospital, Institute of Human Genetics, Kollegiengasse 10, D-07743 Jena, Germany; 2 Research Center of Maternal and Child Health Protection, Mashtots Ave. 22, 0002 Yerevan, Armenia; 3 Center for Human Genetics, Mollstr. 49a, 68165 Mannheim, Germany; 4 Joint Practice of Human Genetics, Friedrichstraße 38/40, 01067 Dresden, Germany; 5 Joint Practice and Cytogenetic Laboratory, Johannisplatz 1, 04103 Leipzig, Germany

## Abstract

**Background:**

Cytogenetically visible unbalanced chromosomal abnormalities (UBCA), reported for >50 euchromatic regions of almost all human autosomes, are comprised of a few megabases of DNA, and carriers are in many cases clinically healthy. It may be speculated, that some of the UBCA may be similar or identical to copy number variants (CNV) of the human genome.

**Results:**

Here we report on a yet unreported cytogenetically visible copy number variant (CNV) in the long arm of chromosome 8, region 8q21.2, detected in three unrelated clinically healthy carriers.

**Conclusion:**

The first description of a cytogenetically visible CNV/UBCA in 8q21.2 shows that banding cytogenetics is far from being outdated. It is a cost efficient, up-to-date method for a single cell specific overview on the whole genome, still prepared to deliver unexpected findings.

## Background

Structural variation of the human genome including large insertions and deletions of DNA, denoted as copy-number variants (CNVs), as well as balanced chromosomal rearrangements, such as inversions, contribute to a major proportion of genetic variance in human [1]. Up to 12% of genome is constituted by CNV, which can arise both meiotically and somatically [2-4]. CNV were identified by array-based approaches and include hundreds of previously undetected structural variants in the human genome [5-7].

The finding of unbalanced chromosomal abnormalities (UBCA) was recently reviewed and summarized from a total of 200 families [8,9]. UBCA usually involve several megabases of DNA, and carriers of such a UBCA are ascertained in most cases either through an abnormal phenotype or adverse reproductive effects [10,11].

Recently, it became possible to connect DNA polymorphism at a molecular genetic with microscopically visible molecular cytogenetic level by using CNV-specific bacterial artificial chromosomes (BACs) as probes for fluorescence in situ hybridization (FISH) [4,7,12]. Applying such a CNV-specific BAC from 8q21.2 we were able to characterize a new chromosomal region involved in a cytogenetic visible UBCA in three healthy persons.

## Results

One female (case 1) and two male (cases 2 and 3) were studied by banding cytogenetics. Cases 1 and two were of Middle-European descent, while case 3 was Japanese. Case 1 was referred for reasons of family planning, as there was mental retardation and cystic fibrosis observed within close relatives. Case 2 was studied due to fertility problems and planned ICSI and case 3 had azoospermia with infertility.

**Figure 1:**
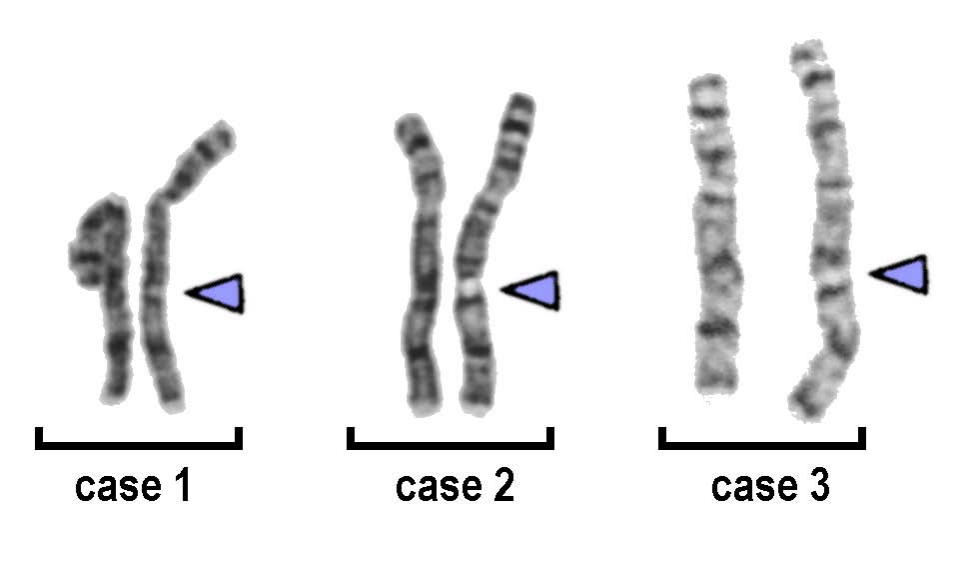
**Result if GTG-banding**. Chromosome banding revealed in three independent cases an enlarged band 8q21.2 (arrowheads).

**Figure 2:**
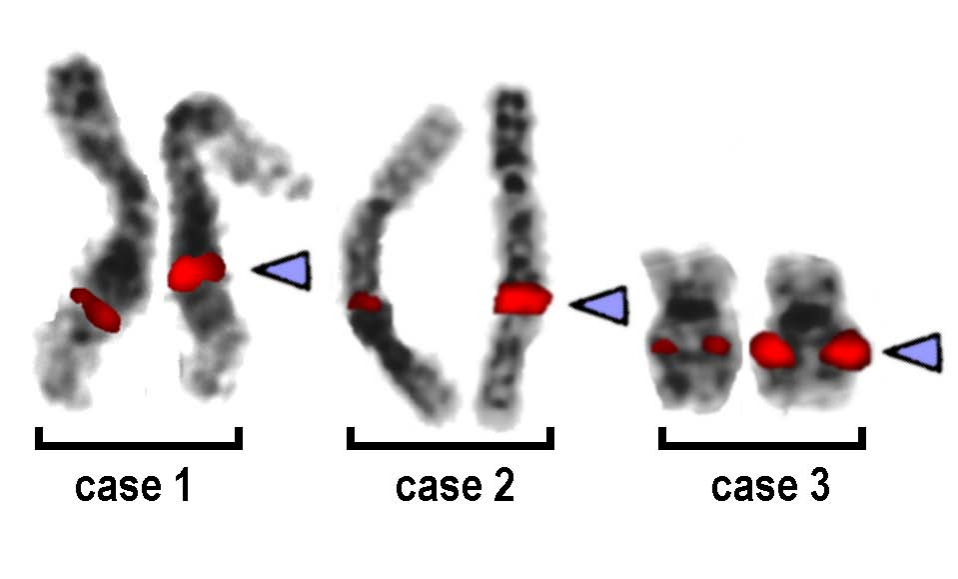
**CNV-specific BAC-FISH-result**. CNV-specific BAC-FISH applying the probe RP11-96G1 at position 86,898,422 to 86,955,420 in 8q21.2 (red signals) revealed a stronger signal on the derivative chromosomes 8 (arrowhead) compared to those signals on the normal chromosomes 8. For case 3 intentionally chromosomes at a low banding resolution are depicted to demonstrate that the CNV is also, maybe even better, visible on short chromosomes.

**Figure 3:**
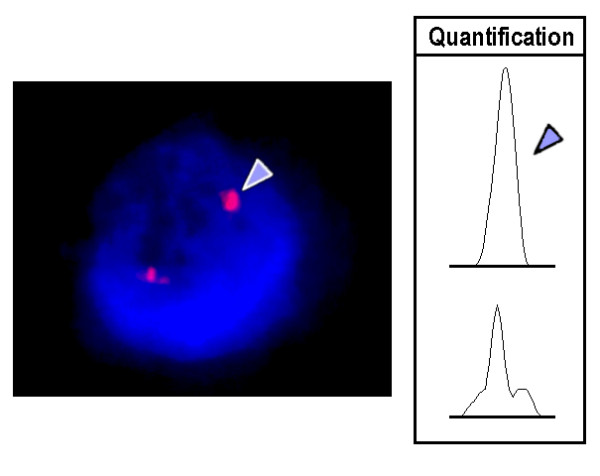
**Scion Image signal intensity measurement**. In interphase it is obvious that there is a CNV but no duplication of the signal RP11-96G1 produces. This is a typical pattern as observed in all up to now studied CNV-specific BACs [4,12]. Applying Scion Image (Scion Corp: http://en.bio-soft.net/draw.html) signal intensity measurement was done, revealing an approximate duplication of signal intensity on the 'derivative' chromosome 8. The arrowhead highlights the larger signal in case 2.

In all three unrelated individuals one chromosome 8 had an abnormal GTG-banding pattern, suggesting a duplication, insertion or inversion (Figure 1). Molecular cytogenetics applying a panel of BAC probes from the corresponding region in 8q21 did not show any aberrant signal patterns (RP11-27N21, RP11-260D13, RP11-317J10, RP11-354A14 - results not shown). However, applying the CNV-spanning BAC RP11-96G1 at position 86,898,422 to 86,955,420 in 8q21.2 revealed a stronger signal on the derivative chromosome 8 (Figure 2). The signal intensities were quantified in 50 to 100 interphase nuclei using the Scion Image (Scion Corp: http://en.bio-soft.net/draw.html) software (Figure 3). This revealed a difference for the two BAC signals BAC RP11-96G1 of ~60% for all three cases (data not shown). Thus, a cytogenetically visible CNV in 8q21.2 is responsible for the aberrant chromosomal banding pattern on the corresponding chromosome 8, which is covered by BAC RP11-96G1.

## Conclusion

A CNV of 8q21.2 covered by the BAC RP11-96G1 was initially described by [5] identified by DNA-array-based methods. In that study, 10 out of 39 healthy individuals had a CNV in the corresponding region. Three of the persons showed a gain, seven a loss of copy numbers in 86.8 to 87.0 Mb. Here for the first time the same CNV was detected as UBCA cytogenetically in three unrelated healthy persons of different ethnic origin. This suggests that the CNV/UBCA in 8q21.2 might be quite frequent in the human population; however, as it is not easily recognized by GTG-banding up to present was simply not recorded in most of the cases.

Interestingly, the CNV/UBCA region was already found to be involved in gene amplification in breast [13] and prostate cancer [14], as well as in aberrant methylation in osteosarcoma [15]. However, the impact of that has to be elucidated in future.

Overall, this description of a cytogenetically visible CNV/UBCA in 8q21.2 shows that banding cytogenetics is still far from being outdated. On the contrary, it is a cost efficient up-to-date method for a single cell specific overview on the whole genome, still prepared to deliver unexpected findings.

## Material and methods

Banding cytogenetic analyses of peripheral blood lymphocytes was performed according to standard protocols [16]. Molecular cytogenetic analysis using BAC-probes was done as previously reported [17]. 20 metaphases per case and BAC probe were analyzed, each. The BACs RP11-27N21, RP11-260D13, RP11-317J10, RP11-354A14 and RP11-96G1 were obtained from BACPAC Chori and the extracted DNA labeled as described in [17].

## Competing interests

The authors declare that they have no competing interests.

## Authors' contributions

MM participated in the molecular cytogenetics studies and drafted the manuscript. FWC, JL and RK provided two of the three studied patients including clinical data, performed cytogenetic analysis and provided GTG-banding figure. CK, CR and HR, provided one of the three studied patients including clinical data, CK and CR performed cytogenetic analysis and provided GTG-banding figures. CS carried out the FISH evaluation with Scion Image-software. EE, KK, MZ and NK participated in and carried out the molecular cytogenetics studies and set up the FISH-probes. TL edited the manuscript. All authors read and approved the final manuscript.
